# A tale of two mixotrophic chrysophytes: Insights into the metabolisms of two *Ochromonas* species (Chrysophyceae) through a comparison of gene expression

**DOI:** 10.1371/journal.pone.0192439

**Published:** 2018-02-13

**Authors:** Alle A. Y. Lie, Zhenfeng Liu, Ramon Terrado, Avery O. Tatters, Karla B. Heidelberg, David A. Caron

**Affiliations:** Department of Biological Sciences, University of Southern California, Los Angeles, California, United States of America; University of Connecticut, UNITED STATES

## Abstract

*Ochromonas* spp. strains CCMP1393 and BG-1 are phagotrophic phytoflagellates with different nutritional strategies. Strain CCMP1393 is an obligate phototroph while strain BG-1 readily grows in continuous darkness in the presence of bacterial prey. Growth and gene expression of strain CCMP1393 were investigated under conditions allowing phagotrophic, mixotrophic, or phototrophic nutrition. The availability of light and bacterial prey led to the differential expression of 42% or 45–59% of all genes, respectively. Data from strain CCMP1393 were compared to those from a study conducted previously on strain BG-1, and revealed notable differences in carbon and nitrogen metabolism between the 2 congeners under similar environmental conditions. Strain BG-1 utilized bacterial carbon and amino acids through glycolysis and the tricarboxylic acid cycle, while downregulating light harvesting and carbon fixation in the Calvin cycle when both light and bacteria were available. In contrast, the upregulation of genes related to photosynthesis, light harvesting, chlorophyll synthesis, and carbon fixation in the presence of light and prey for strain CCMP1393 implied that this species is more phototrophic than strain BG-1, and that phagotrophy may have enhanced phototrophy. Cellular chlorophyll *a* content was also significantly higher in strain CCMP1393 supplied with bacteria compared to those without prey. Our results thus point to very different physiological strategies for mixotrophic nutrition in these closely related chrysophyte species.

## Introduction

Mixotrophic protists are single-celled eukaryotes capable of acquiring carbon, nutrients or energy via more than one trophic mode. These organisms span multiple phyla and are commonly found in a broad array of environments [1]. Their prevalence, and even dominance in certain communities, make them significant players in the flow of energy and nutrients, and increasingly more studies exhort the importance of including mixotrophy in biogeochemical models and ecosystem studies [2–4].

The nutritional strategies of mixotrophic (phagotrophic) phytoflagellates exhibit great variety, with a spectrum of mixotrophic nutrition ranging from nearly pure phototrophy to nearly pure heterotrophy [5]. For example, some algae may ingest prey to obtain macronutrients (e.g. nitrogen or phosphorus) or essential trace growth factors (e.g. iron or vitamins), but largely use phototrophy to produce organic carbon for growth. Conversely, predominantly heterotrophic phytoflagellates may rely on photosynthesis only for survival when the abundance of prey is low. The availability of resources (e.g. light or prey) in the environment can affect which nutritional mode(s) is performed by mixotrophs, and such nutritional flexibility complicates our ability to define their functional roles in natural communities. Improving our understanding of the nutritional strategies and metabolism of mixotrophs will enable a better incorporation of mixotrophy into food web or biogeochemical models.

*Ochromonas* is a genus of mixotrophic chrysophytes consisting of > 100 described species that exhibit a variety of nutritional strategies [6]. Many *Ochromonas* species, such as *Ochromonas danica*, have been found to be predominantly phagotrophic as they can grow in continuous darkness as long as bacterial prey is available (e.g. [7–9]). However, other *Ochromonas* species rely more on phototrophy, and some even have an absolute requirement for light [10–12]. We previously conducted an investigation of the gene expression of a freshwater *Ochromonas* sp. (strain BG-1) under conditions that induced phagotrophic (only prey available), mixotrophic (light and prey available), and phototrophic (only light available) nutritional modes [13]. This *Ochromonas* species is predominantly phagotrophic since its growth rate in continuous darkness with bacterial prey was equivalent to that in continuous light with prey. Transcriptomic results were in agreement with the alga’s growth as the availability of light had a relatively small effect (differential expression of 8% of all genes) on the alga’s gene expression compared to the effect of the availability of prey (differential expression of 59% of all genes). However, a number of genes related to photosynthesis and lysosomal proteases were upregulated in the presence of light. An analysis of the expression of genes related to major carbon metabolic pathways (e.g. glycolysis) indicated that this species had a strong reliance on bacterial carbon while prey was available, but increased in photosynthetic capabilities when prey was depleted.

In this study, we performed a similar experiment using a marine *Ochromonas* sp. strain CCMP1393 that has a different nutritional strategy than strain BG-1. This marine species is obligately phototrophic as it does not increase in population abundance in continuous darkness even in the presence of bacterial prey. We analyzed the gene expression of strain CCMP1393 under different conditions of light and bacterial prey availability, and found that strain CCMP1393 appeared to have high photosynthetic activities even in the presence of prey. Our results implied that there are synergistic effects between phagotrophy and photosynthesis in this alga, as the presence of both light and prey resulted in increased growth rates and upregulation of genes related to photosynthesis. Transcriptomic results thus revealed considerable differences in the physiologies between 2 species of *Ochromonas* and demonstrated the diversity of mixotrophic behaviors and metabolism among mixotrophic species even within a genus.

## Materials and methods

### Organisms and cultures

*Ochromonas* sp. strain CCMP1393 was obtained from the National Center for Marine Algae and Microbiota culture collection (formerly the CCMP). The alga was subsequently rendered axenic using antibiotics: 100 mg of penicillin and 50 mg of streptomycin dissolved in 10 ml of ultrapure water (Barnstead GenPure xCAD Plus, Thermo Fisher Scientific, Waltham, USA), combined with 20 mg of chloramphenicol dissolved in 0.5 ml of 95% ethanol [7]. Sterility tests in this study were performed by adding 5 ml aliquots of culture to 7 ml of Difco marine broth 2216 (Becton Dickinson, Franklin Lakes, USA) and 0.3% yeast extract. Sterility tests were performed periodically to check for growth of live bacteria or fungi. Cultures were deemed sterile if no growth of such contaminants was observed after two weeks.

Axenic cultures of strain CCMP1393 were maintained in a modified K medium (S1 Table) made with aged (> 3 months) and 0.2 μm filtered seawater from the San Pedro Ocean Time-series station (33° 33’N, 118° 24’W). Heat-killed bacteria (HKB) were provided to cultures to support growth. The bacterial strain used to produce HKB was isolated from a bacterized culture of freshwater *Ochromonas* sp. strain BG-1 by streaking onto a 1.5% agar plate with 0.5% yeast extract and 0.5% tryptone. A single bacterial colony was picked and grown for ~4 days in 0.5% yeast extract and 0.5% tryptone broth. The bacterial clonal culture was heat-killed at 70°C for 30 minutes, and then rinsed and concentrated through 3 rounds of centrifugation (10,000 g for 15 minutes; Sorvall RC5C plus, Thermo Fisher) and resuspension in sterile ultrapure water.

### Experimental setup

**Fig 1:**
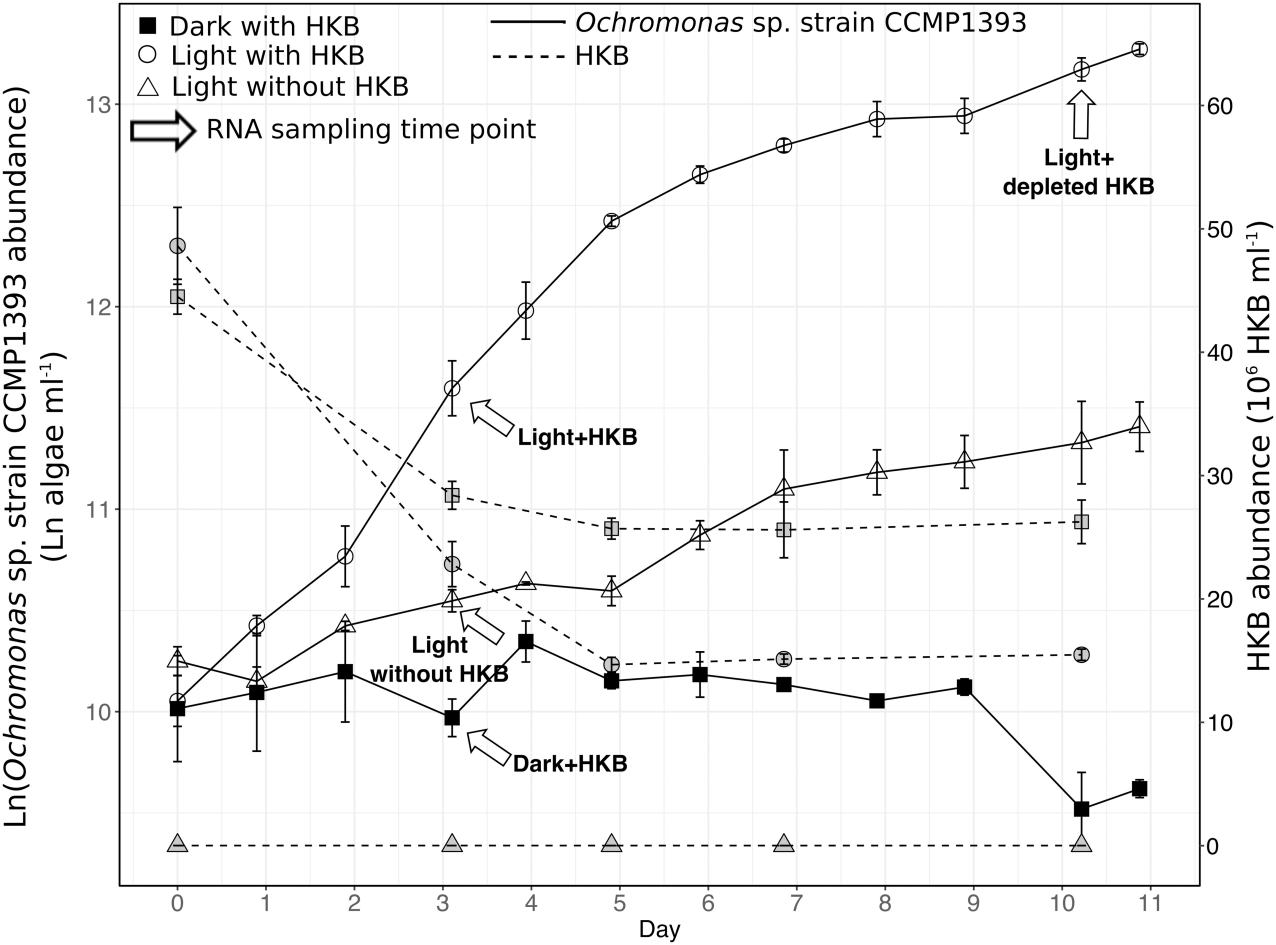
Abundances of *Ochromonas* sp. strain CCMP1393 and HKB. Average abundances (± SD; *n* = 2 for Dark with HKB and Light without HKB; *n* = 4 for Light with HKB) of *Ochromonas* sp. strain CCMP1393 and absolute abundance of heat-killed bacteria (HKB) in different experimental treatments. The slopes of the abundance curves reflect the algae’s growth rates. Arrows indicate samples collected on day 3 and day 10 for RNA extraction.

Batch cultures of *Ochromonas* sp. strain CCMP1393 were grown in Fernbach flasks under different light and HKB availability at 20°C. Cultures were grown in modified K medium on an orbital shaker table (Model 3500, VWR, Radnor, USA) at 60 rpm to prevent the settling of HKB, and placed in either continuous light (20 μEinsteins m^-2^ s^-1^; QSL-100 sensor with QSP-170 deckbox, Biospherical Instruments Inc., San Diego, USA) or continuous darkness (flasks were wrapped with aluminum foil). An algal inoculum culture grown under continuous light was used to inoculate all experimental cultures. Experimental cultures with a total volume of 1.3 L were inoculated at a starting algal abundance of ~ 2.4 x 10^4^ algae ml^-1^. A one-time dose of HKB was added to all culture vessels (final concentration of ~ 5 x 10^7^ HKB ml^-1^) receiving HKB. There were 3 experimental treatments: dark with HKB (duplicates); light with HKB (quadruplicates); and light without HKB (duplicates). Samples for RNA extraction were collected on day 3 from duplicate cultures of all 3 conditions when HKB were still present at abundances that allowed their ingestion by the alga (Fig 1). The remaining 2 replicates of the light with HKB cultures were harvested for RNA on day 10, by which time HKB grazing activity had ceased (Fig 1). Sterility tests were performed on days 0, 3, 7, and 11.

A 400 ml aliquot was collected from each replicate culture for RNA extraction, and immediately split into 2 technical replicates (200 ml) that were processed separately. Samples were concentrated by centrifugation (5,000 g for 10 min; Allegra 25R Centrifuge, Beckman Coulter, Brea, USA). The pellet was preserved in 0.75 ml of RNAlater (Sigma-Aldrich, St. Louis, USA) immediately after centrifugation, and placed at -80°C until further processing. RNA was extracted using a RNeasy Plant Mini Kit (Qiagen, Venlo, NL), and the extracts were subsequently treated with DNase (Sigma-Aldrich) for DNA removal. Total RNA was finally cleaned and concentrated using a RNA Clean & Concentrator kit (Zymo Research, Irvine, USA), and sent to the University of Southern California’s Epigenome Center for sequencing on an Illumina NextSeq 500 (V2 chemistry; 75 bp paired-ends). A starting mass of 1 μg of total RNA was used, and polyA selection was carried out using Illumina Truseq V2 polyA beads according to manufacturer’s instructions, except that an extra round of purification using the same kit was carried out to further deplete samples of RNA from HKB. Material enriched for polyA was then introduced into the Kapa Biosystems stranded RNA-seq library prep kit (Kapa Biosystems, Wilmington, USA) at the elute-fragmentation-priming step and taken through the protocol following manufacturer’s instructions. A total of 10 PCR cycles were performed. Libraries were visualized by Bioanalyzer analysis (Agilent Technologies, Santa Clara, USA) and quantified by qPCR (Illumina Library Quantification Kit, Kapa Biosystems, Wilmington, USA).

Samples for determining algal and bacterial cell abundances were taken periodically to determine the growth rates of strain CCMP1393 and rates of HKB ingestion by the alga. A 1 ml aliquot was preserved with acid Lugol’s solution (final concentration 5%) to estimate algal abundance, and enumerated using a Palmer-Maloney counting chamber at 200 x on a compound light microscope (BX51; Olympus, Waltham, USA). HKB abundance was estimated by preserving 3 ml aliquots with formaldehyde (final concentration 1%), which was then filtered onto a 0.2 μm black polycarbonate filter (25 mm, Whatman, Maidstone, UK), stained with 4’ 6-diamidino-2-phenylindole dihydrochloride (DAPI; 30 μg; Sigma-Aldrich), and enumerated using a compound microscope equipped with epifluorescence illumination (BX51; Olympus). Ingestion rates of the alga were calculated using the formula:

$$\left({\left[\text{HKB}\right]}_{\text{final}}–\;{\left[\text{HKB}\right]}_{\text{initial}}\right)/\left(\left[\text{Average}\;\text{algal}\;\text{abundance}\right]\;\times \text{Change}\;\text{in}\;\text{time}\right)$$

in which average algal abundance was calculated as:

$$\left({\left[\text{Algae}\right]}_{\text{final}}-{\left[\text{Algae}\right]}_{\text{initial}}\right)/\left(\text{LN}\left({\left[\text{Algae}\right]}_{\text{final}}/{\left[\text{Algae}\right]}_{\text{initial}}\right)\right).$$

Cellular chlorophyll *a* was determined for the inoculum culture, as well as from all experimental cultures on days 1, 3, 5, 7, and 10. Aliquots (25–50 ml) were filtered onto GF/F filters (25 mm, Whatman) and samples were stored at -80°C until extraction. Acetone (100%, Sigma-Aldrich) was used to extract chlorophyll *a* for 24 h in the dark at -20°C, and fluorescence was measured on a fluorometer (Trilogy; Turner Designs, San Jose, USA) using a non-acidification method [14].

### Transcriptome processing

The transcriptome of *Ochromonas* sp. strain CCMP1393 was assembled *de novo* from the 16 libraries (4 treatments x 2 replicates x 2 technical replicates). Assembly was performed using the software Trinity with a k-mer size of 25 bp [15], and script align_and_estimate_abundance.pl included in Trinity (with RSEM [16] and bowtie [17]) were used to estimate fragment per kilobase of transcript per million reads (fpkm) values and percentages of isoform support. Transcripts with < 1% isoform support and < 1 fpkm in all libraries were discarded. TransDecoder [18] was used to identify potential coding regions within reconstructed transcripts, and CD-HIT-EST [19] was used to remove identical redundant coding genes. Putative genes were annotated using protein databases, including GenBank, Tigrfam, and Pfam (e-value cutoff: 10^−5^). The KEGG Automatic Annotation Server (KAAS) was used to provide additional information on the metabolic process(es) associated with each gene [20]. Gene annotation for genes discussed in detail in this study was further manually curated based on the combination of results from all 3 protein databases mentioned above. Fpkm was used to normalize expression among the 16 libraries. If the fragment count of a gene was 0 in a library, then the fragment count was replaced with 1 to allow downstream processing e.g. the calculation of fold changes. Genes that did not have fpkm ≥ 1 in at least 2 or more libraries were removed from the transcriptome to minimize potential sequencing errors or assembly artifacts. Assembled sequences were deposited in the NCBI Sequence Read Archive under accession number SRP115947.

The statistical software package edgeR was used to perform pairwise comparisons to identify genes that were differentially expressed between 2 treatments (false-discovery rate < 0.05; [21]). There were 3 pairwise comparisons that investigated the effect of light or HKB: 1 with the availability of light as the variable factor (i.e. Dark+HKB vs. Light+HKB), and 2 with the availability of HKB as the variable factor (i.e. Light+depleted HKB vs. Light+HKB; Light without HKB vs. Light+HKB).

### Phylogeny of *Ochromonas*

An unrooted phylogenetic tree of *Ochromonas* species supplemented with additional chrysophytes and synurophytes was constructed using 18S rRNA gene sequences retrieved from GenBank or the Marine Microbial Eukaryote Transcriptome Sequencing Project (MMETSP) database. A segment of 1,677 positions on the 18S rRNA gene was selected through a combination of Gblocks [22] and manual editing to use for tree building using MUSCLE [23]. Approximate maximum likelihood trees were constructed using FastTree v2.1 [24] in ‘accurate mode’ (-mlacc 2-slowmmi) with the general time reversible model and pseudocounts (Number of bootstrap = 1,000).

## Results

### Growth of strain CCMP1393 under different conditions

The growth of *Ochromonas* sp. strain CCMP1393 was highest in the presence of both light and HKB (Table 1; Fig 1). Algae kept in continuous darkness with HKB (Dark with HKB curve in Fig 1) did not increase in abundance, while those kept in continuous light without receiving any HKB (Light without HKB curve in Fig 1) at the start of the experiment had a slow growth rate of 0.11 d^-1^. In contrast, algae with both light and HKB (Light with HKB curve in Fig 1) had a growth rate of 0.48 d^-1^ over the first 5 days of the experiment, but decreased to 0.14 d^-1^ after day 5.

**Table 1:** Average growth rate (± standard deviation) and grazing rates (± standard deviation) of *Ochromonas* sp. strain CCMP1393 in different treatments.

Treatment	Growth of alga		Grazing rate on HKB	
		Growth rate (d^-1^)		Grazing rate (HKB alga^-1^ h^-1^)
Dark with HKB	Day 0 –Day 11	-0.03 ± 0.02	Day 0 –Day 3	10 ± 2
Light with HKB	Day 0 –Day 7	0.40 ± 0.02	Day 0 –Day 3	6 ± 1
	Day 7 –Day 11	0.12 ± 0.01		
Light without HKB	Day 0 –Day 11	0.11 ± 0.01	NA	NA

The abundance of HKB decreased over the first 5 days of the experiment for both the light and dark treatments supplied with HKB (Fig 1). Bacterial grazing rates during the first 3 days were similar in these 2 treatments at 6±1 bacteria alga^-1^ h^-1^ and 10±2 bacteria alga^-1^ h^-1^ for the treatment in continuous light and darkness respectively (one-way ANOVA *p* > 0.05; Table 1), despite the lack of algal growth in the dark. Grazing by the algae appeared to cease after day 5 in both treatments as there was no further significant decrease in bacterial abundances. DAPI stained samples for enumerating HKB abundances revealed that the remaining HKB from day 5 onwards were strongly aggregated compared to samples from day 0 and day 3. This was presumably due to mucus released by *Ochromonas* sp., and the aggregated HKB was likely less accessible as prey for the algae.

**Fig 2:**
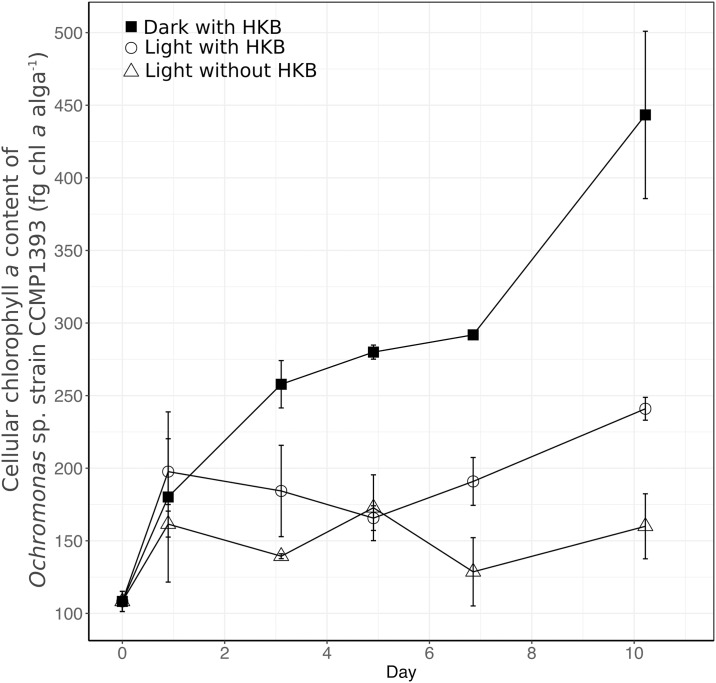
Cellular chlorophyll content of *Ochromonas* sp. strain CCMP1393. Average cellular chlorophyll content (± SD) of *Ochromonas* sp. strain CCMP1393 in different experimental treatments over the course of the experiment.

Cellular chlorophyll *a* concentrations changed significantly between treatments during the experiment. Chlorophyll *a* contents in all treatments increased slightly between day 0 (taken from the inoculum) and day 1 (Fig 2). Algae kept in continuous darkness increased in cellular chlorophyll *a* content throughout the course of the experiment despite their lack of increase in abundance, resulting in a significantly higher final (day 10) cellular chlorophyll *a* content in algae kept in dark with HKB than that in all other treatments (443 ± 57 fg alga^-1^; Two-way ANOVA, *p* < 0.001). Chlorophyll *a* content of algae incubated in continuous light with HKB (241 ± 8 fg alga^-1^) was significantly higher than algae maintained at the same light intensity but without receiving any HKB (Light without HKB treatment; 160 ± 22 fg alga^-1^; Two-way ANOVA, *p* < 0.05).

### Overview of transcriptome and differentially expressed genes of strain CCMP1393

The *de novo* assembly of *Ochromonas* sp. strain CCMP1393 yielded a transcriptome size of 34 Mbp. It consisted of 27,227 genes, but the removal of genes that did not have fpkm ≥ 1 in at least 2 or more libraries reduced the total number of genes to 19,692.

The availability of light and HKB yielded comparable numbers of differentially expressed genes (Table 2). A pairwise comparison of the Dark+HKB vs. Light+HKB treatments yielded a total of 8,323 (42% of 19,692 genes) differentially expressed genes (left 2 columns of Table 2), while comparisons between conditions with and without HKB (i.e. Light+depleted HKB vs. Light+HKB; Light without HKB vs. Light+HKB) yielded a total of 8,763 or 11,568 genes (an average of 52% of 19,692 genes) that were differentially expressed (right 4 columns of Table 2).

**Table 2:** Number of differentially expressed genes (false discovery rate < 0.05) in different pairwise comparisons of treatments in the *Ochromonas* sp. strain CCMP1393 experiment.

	Effect of light		Effect of HKB			
	Dark+HKB vs. Light+HKB		Light+depleted HKB vs. Light+HKB		Light without HKB vs. Light+HKB	
	Upregulated in Dark+HKB	Upregulated in Light+HKB	Upregulated in Light+depleted HKB	Upregulated in Light+HKB	Upregulated in Light without HKB	Upregulated in Light+HKB
**Total genes** (19,692 genes)	3,902	4,421	4,398	4,365	5,654	5,914
**Photosynthesis-related**						
Antenna proteins KO00196 (10 genes)	2	4	2	6	3	5
Carotenoid biosynthesis KO00906 (8 genes)	3	1	2	3	2	4
Photosynthesis KO00195 (17 genes)	2	8	2	11	3	10
**Tetrapyrrole synthesis** (43 genes)	10	14	5	23	4	29
**Carbon metabolism**						
PPP (14 genes)	2	10	2	9	3	8
Calvin cycle (7 genes	2	3	0	5	0	5
Glycolysis (29 genes)	9	12	7	11	8	12
PEP to TCA (25 genes)	2	20	16	1	18	1
TCA cycle (32 genes)	2	23	6	14	6	17
**Nitrogen metabolism** (18 genes)	6	3	4	9	6	9

### Genes associated with light harvesting and photosystems

**Fig 3:**
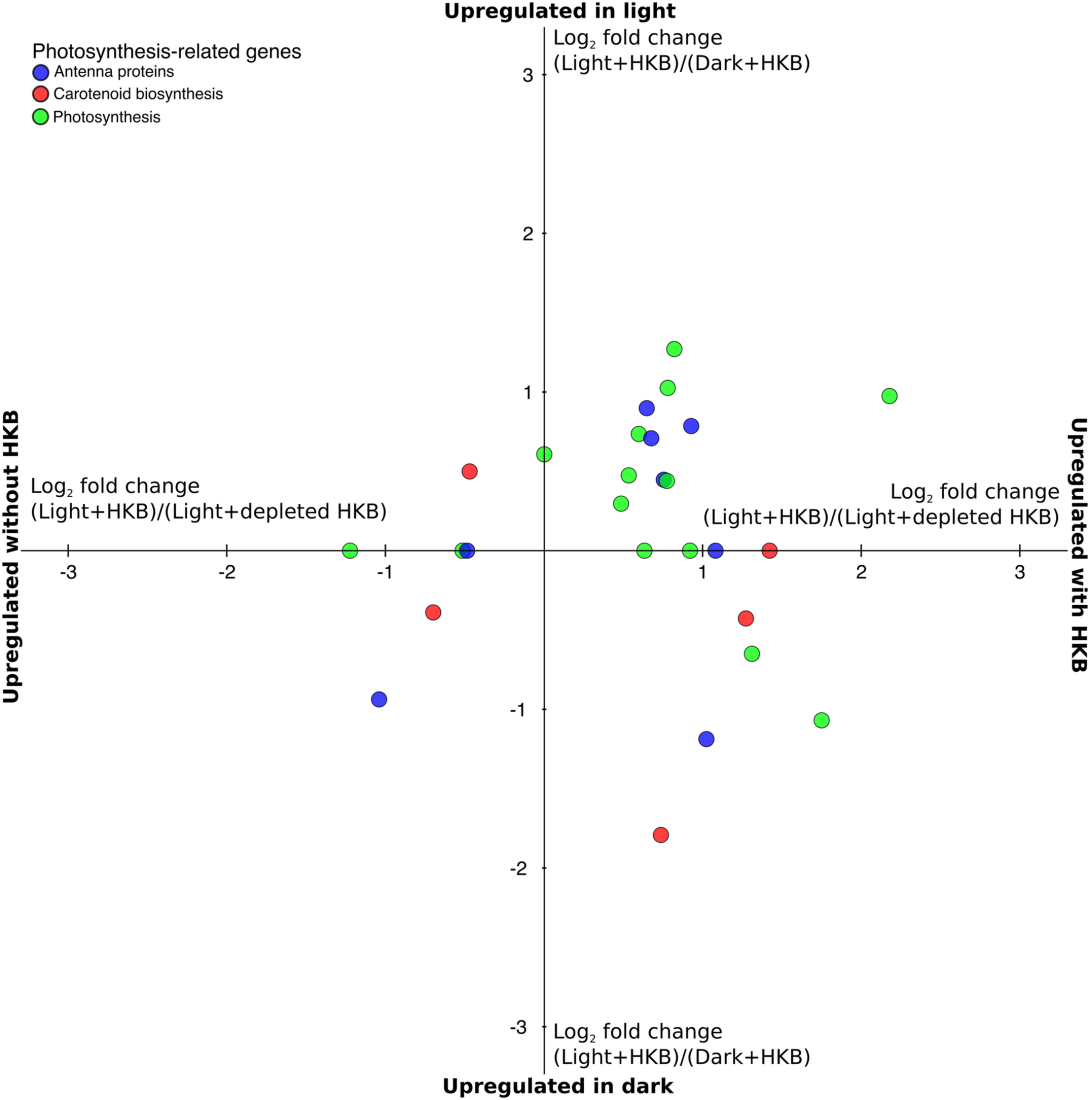
Fold change of differentially expressed genes related to photosynthesis of *Ochromonas* sp. strain CCMP1393. Average log_2_ fold change of differentially expressed genes related to photosynthesis of *Ochromonas* sp. strain CCMP1393 in the pairwise comparisons: Light+HKB vs. Dark+HKB (y-axis); and Light+HKB vs. Light+depleted HKB (x-axis). Values above the x-axis indicate genes upregulated in the Light+HKB treatment compared to the Dark+HKB treatment, while values to the right of the y-axis indicate genes upregulated in the Light+HKB treatment compared to the Light+depleted HKB treatment. Values on the x-axis indicate genes not differentially expressed between the Dark+HKB and Light+HKB treatments (i.e. expression of these were only affected by the availability of prey), while values on the y-axis indicate genes not differentially expressed between the Light+HKB and Light+depleted HKB treatments (i.e. expression of these were only affected by the availability of light). Colors indicate the KEGG ortholog group of the gene.

A total of 35 genes related to photosynthesis were identified in the transcriptome of strain CCMP1393, and most had a positive response to the presence of HKB (data points to the right of the y-axis of Fig 3). Genes associated with photosynthesis and light harvesting were defined as those involved in 3 KEGG ortholog groups: photosynthesis antenna proteins (KO00196), carotenoid biosynthesis (KO00906), and photosynthesis (KO00195). The majority of genes for antenna proteins were upregulated in the presence of HKB (right 4 columns of Table 2). The expression of genes for carotenoid synthesis were not consistently affected by the availability of HKB, with 50% and 38% of them upregulated in algae from Light+HKB compared to Light+depleted HKB (middle 2 columns of Table 2) and Light+HKB compared Light without HKB respectively (right 2 columns of Table 2). Over half of the genes for photosystem proteins (under the KOG group ‘photosynthesis’) had higher expression in the presence of HKB when compared to the treatment without HKB or with depleted HKB (right 4 columns of Table 2). Most of the genes for proteins in the photosystems were also upregulated in the presence of both light and HKB (data points in the upper right quadrant of Fig 3).

**Fig 4:**
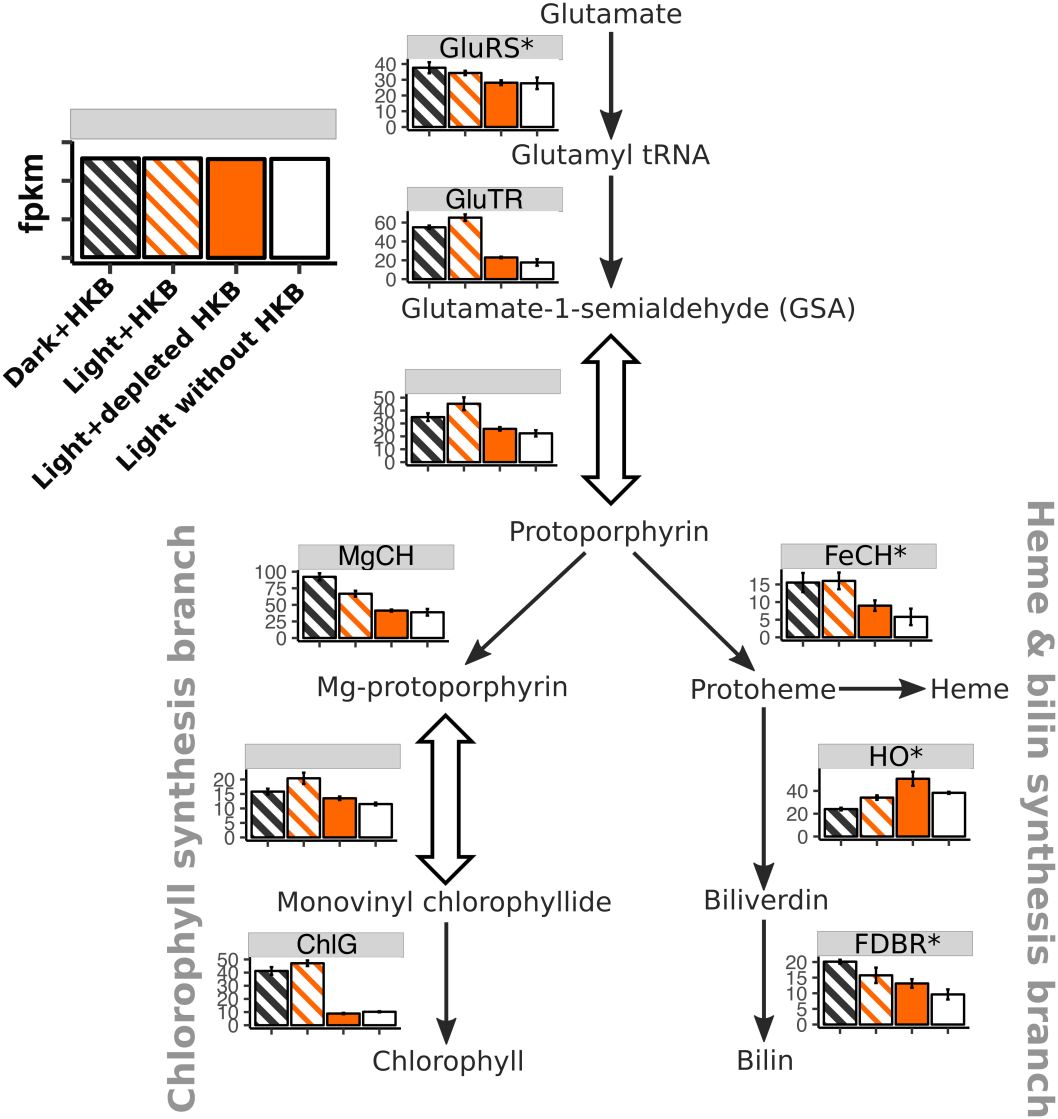
Heatmap expression of genes associated with tetrapyrrole synthesis for *Ochromonas* sp. strain CCMP1393. Average fragment per kilobase of transcript per million reads (fpkm; ± SD) of genes associated with tetrapyrrole synthesis for *Ochromonas* sp. strain CCMP1393 in different treatments. An asterisk next to the enzyme abbreviation (Refer to S2 Table for the full name of each enzyme) indicates there were multiple paralogs for the enzyme, in which case the fpkm was calculated as the geometric mean of all paralogs. Block arrows indicate pathways that involve multiple enzymes, and fpkm were summarized as the geometric mean of all genes and their paralogs. Striped bars indicate treatments with HKB.

Genes related to tetrapyrrole and thus chlorophyll synthesis also had a positive response to the presence of HKB (Fig 4). More than half (53%– 67%) of these genes were upregulated in the Light+HKB treatment compared to the treatments without HKB or depleted HKB (right 4 columns of Table 2). Key genes related to chlorophyll synthesis, including those coding for glutamyl-tRNA reductase (GluTR), magnesium chelatase (MgCH), and chlorophyll synthase (ChlG) [25], were all consistently upregulated in the presence of HKB when compared to conditions without HKB (i.e. Light+depleted HKB vs. Light+HKB; Light without HKB vs. Light+HKB; Fig 4) except for 1 paralog of MgCH.

### Major carbon metabolic pathways

**Fig 5:**
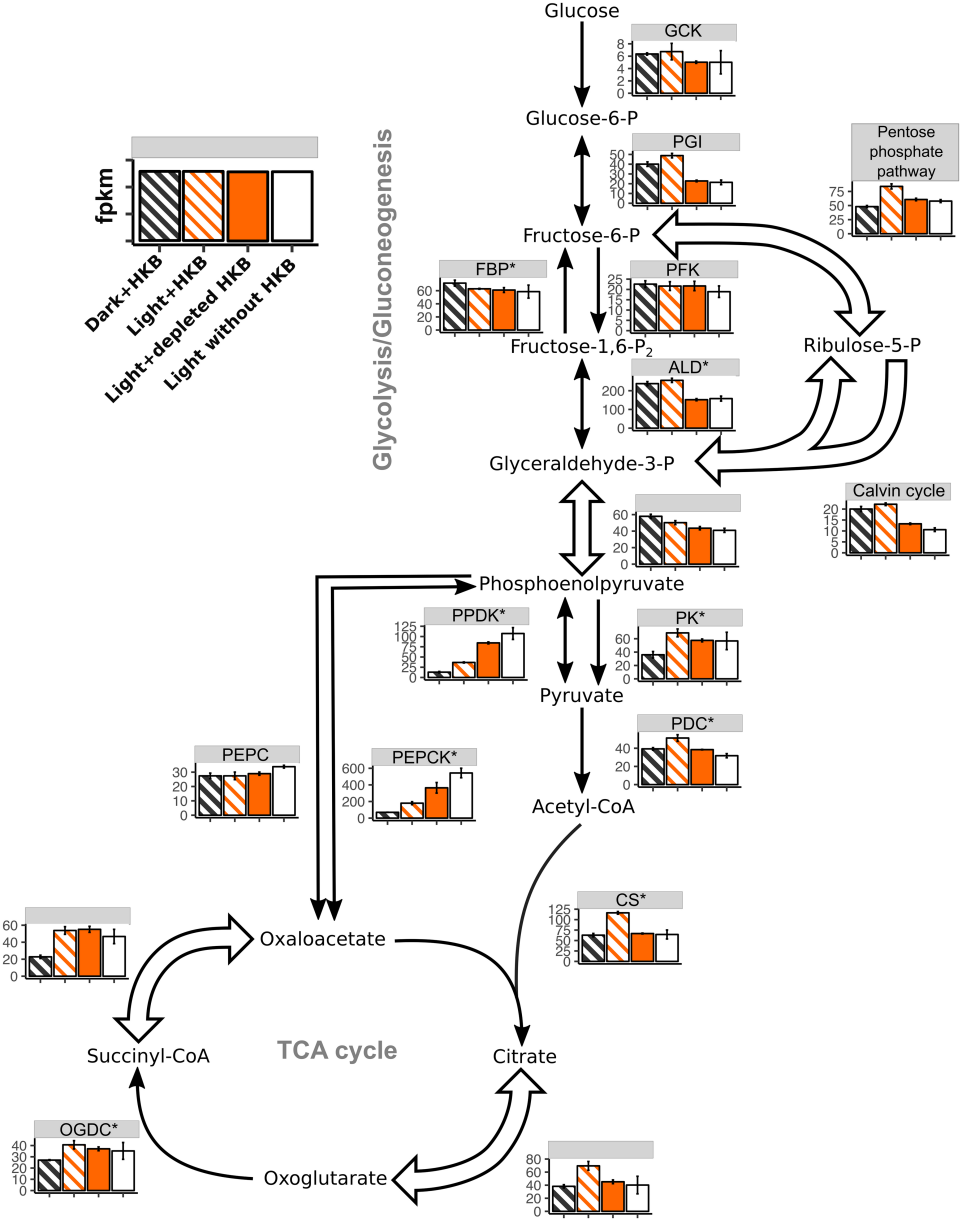
Heatmap expression of genes associated with major carbon metabolism for *Ochromonas* sp. strain CCMP1393. Average fragment per kilobase of transcript per million reads (fpkm; ± SD) of genes associated with major carbon metabolism for *Ochromonas* sp. strain CCMP1393 in different treatments. An asterisk next to the enzyme abbreviation (Refer to S2 Table for the full name of each enzyme) indicates there were multiple paralogs for the enzyme, in which case the fpkm was calculated as the geometric mean of all paralogs. Block arrows indicate pathways that involve multiple enzymes, and fpkm were summarized as the geometric mean of all genes and their paralogs. Only genes for enzymes exclusive to the Calvin cycle (i.e. ribulose-1,5-bisphosphate carboxylase/oxygenase, sedoheptulose bisphosphatase, and phosphoribulokinase) were categorized as ‘Calvin cycle’. Striped bars indicate treatments with HKB.

There were 107 genes associated with glycolysis/gluconeogenesis, nonoxidative pentose phosphate pathway (PPP), Calvin cycle, and tricarboxylic acid (TCA) cycle. The availability of light and HKB led to the differential expression of 79% and an average of 70% of these genes, respectively (Table 2). Genes exclusively associated with the Calvin cycle (i.e. ribulose-1,5-bisphosphate carboxylase/oxygenase (Rubisco), sedoheptulose bisphosphatase, and phosphoribulokinase) were not affected by the availability of light, as their overall expression was comparable between the Dark+HKB and Light+HKB treatments (Fig 5). The presence of HKB led to the upregulation of > 40% of genes in the PPP, Calvin cycle, and TCA cycle (right 4 columns of Table 2), but the expression of genes for phosphoenolpyruvate carboxykinase (PEPCK) and pyruvate phosphate dikinase (PPDK) that connects phosphoenolpyruvate to the TCA cycle were downregulated in the presence of HKB (Fig 5).

### Major nitrogen metabolic pathways

**Fig 6:**
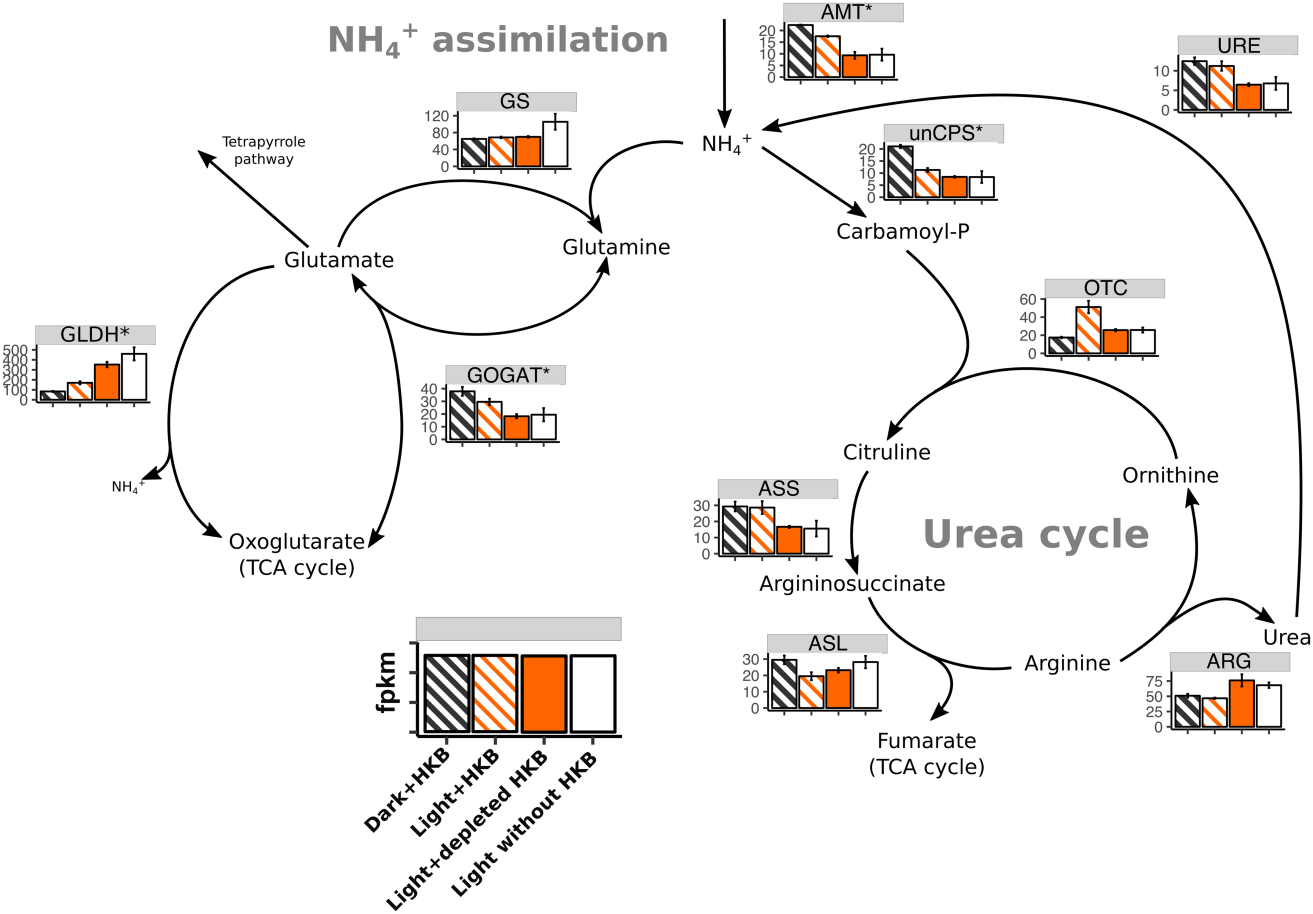
Heatmap expression of genes associated with ammonium assimilation and the urea cycle for *Ochromonas* sp. strain CCMP1393. Average fragment per kilobase of transcript per million reads (fpkm; ± SD) of genes associated with ammonium assimilation and the urea cycle for *Ochromonas* sp. strain CCMP1393 in different treatments. An asterisk next to the enzyme abbreviation (Refer to S2 Table for the full name of each enzyme) indicates there were multiple paralogs for the enzyme, in which case the fpkm was calculated as the geometric mean of all paralogs. Striped bars indicate treatments with HKB.

A total of 18 genes associated with ammonium assimilation and the urea cycle were identified in the transcriptome of *Ochromonas* sp. strain CCMP1393, and 50% of them were upregulated when HKB were available (right 4 columns of Table 2). Genes associated with the uptake and assimilation of nitrate (i.e. nitrate transporter, nitrite transporter, nitrate reductase, and nitrite reductase) were not found in the transcriptome. Their absence was manually confirmed by BLAST searches of homologs from other organisms against the entire transcriptome. The overall expression of genes for ammonium transporters (AMT) were highest in treatments with bacterial prey (Fig 6), as were genes for urease (URE) and glutamine oxoglutarate aminotransferase (GOGAT). The gene expression of glutamate dehydrogenase (GLDH), in contrast, was highest when the alga was growing slowly with light but no HKB (i.e. Light without HKB or Light+depleted HKB).

### Phylogeny of *Ochromonas*

**Fig 7:**
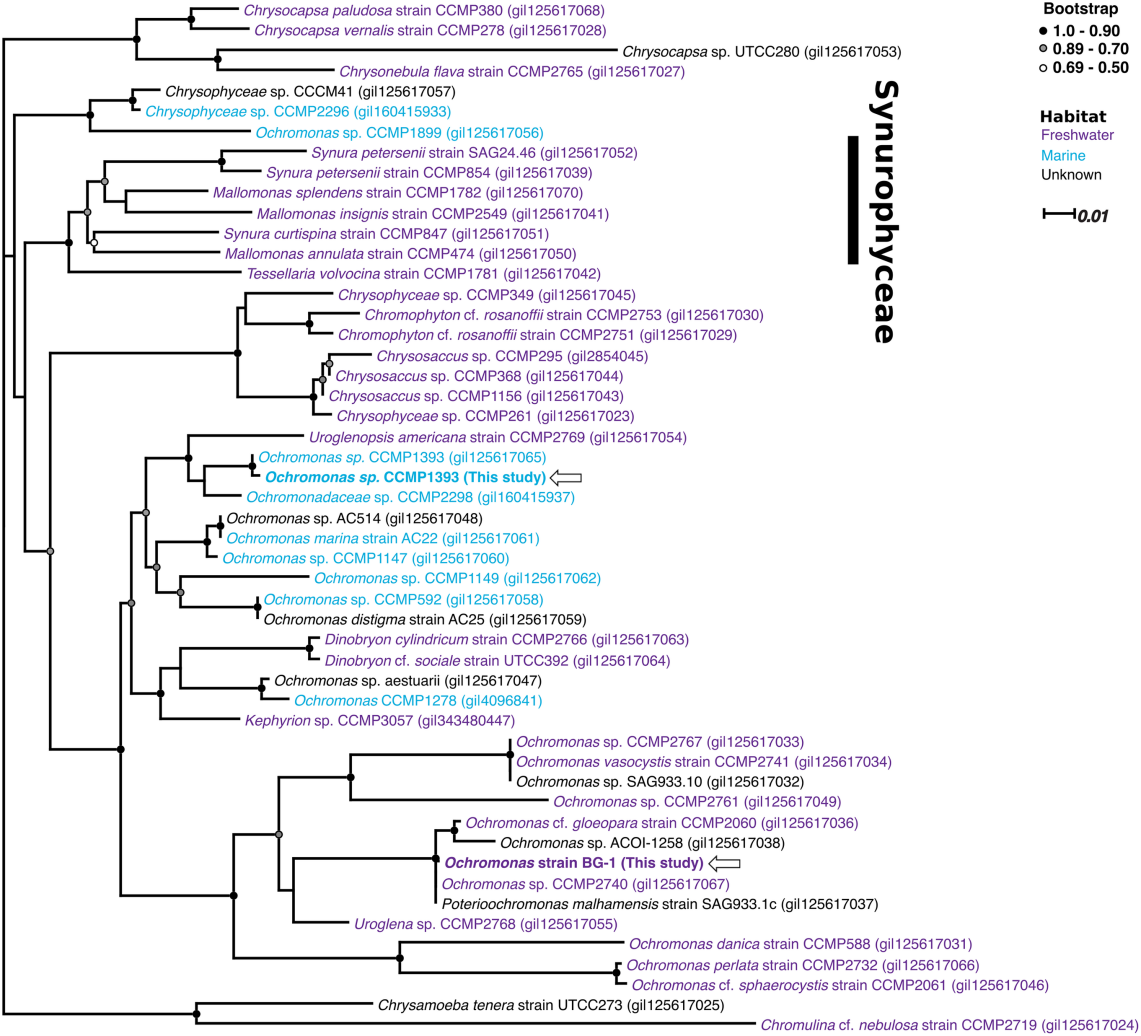
Phylogenetic tree of *Ochromonas* and other closely related chrysophyte species. Unrooted approximate maximum likelihood phylogenetic tree of 18s rRNA genes obtained from transcriptomes of *Ochromonas* spp. strains CCMP1393 and BG-1 (indicated by arrows) and other sequences available from public databases. Sequences are identified by species name and its accession number. Color of the species name indicate the habitat of the scpecies: freshwater (blue), marine (purple) and unknown (black). The tree was constructed from aligned sequences over 1,677 bp length with 1,000 iterated bootstraps. Bootstrap at nodes is represented by filled black circles (1.00–0.90), grey circles (0.89–0.70) or white circles (0.69–0.50); nodes with support < 0.50 were not indicated.

*Ochromonas* is a polyphyletic genus. A phylogenetic tree of species within this genus and other closely related taxa indicated that the marine *Ochromonas* sp. strain CCMP1393 is closely related to several other marine *Ochromonas* spp. and a few freshwater chrysophytes (e.g. *Dinobryon*) than it is to freshwater *Ochromonas* species, including strain BG-1 (Fig 7).

## Discussion

The genus *Ochromonas* is large and polyphyletic, a consequence of the fact that its defining morphology (i.e. naked single cells with 2 heterodynamic flagella and chloroplasts) is a common feature found in several phylogenetically distinct groups [6]. *Ochromonas* spp. strains CCMP1393 and BG-1 are not closely related (Fig 7), and a comparison of the results from this study and Lie *et al*. [13] reveals very different nutritional strategies and physiologies between these two mixotrophic chrysophytes.

### Nutritional strategies of *Ochromonas* sp. strain CCMP1393 compared to strain BG-1

The 2 strains of *Ochromonas* spp. exhibited different resource requirements for optimal growth. Strain CCMP1393 required both light and prey to achieve maximal growth rates and did not increase in population abundance without the presence of light (Fig 1). Conversely, light was not required for the growth of strain BG-1 if prey was available, and the presence of light had no measurable effect on growth rate when prey were present (Fig. 1 in [13]). Hence, phototrophy in *Ochromonas* sp. strain BG-1 appears to serve as a survival mechanism that might prevent starvation in the absence of sufficient prey. Many *Ochromonas* species investigated in past studies have also been shown to be predominantly phagotrophic, similar to strain BG-1, and the abundances of those species increased rapidly in continuous darkness as long as prey were available [7,26–29]. Nonetheless, some marine *Ochromonas* species, such as *Ochromonas minima*, have been shown to be incapable of growth without light like strain CCMP1393 [10–12,30]. An inspection of genes that were differentially expressed in the Dark+HKB vs. Light+HKB in this study revealed upregulation of genes related to ribosomes, purine and pyrimidine metabolism, as well as DNA replication in the presence of light. However, it is unclear what essential growth factor or process the obligately phototrophic strain CCMP1393 requires from light.

Photosynthesis was more than just a survival mechanism for *Ochromonas* strain CCMP1393 as this species increased in abundance photosynthetically when bacterial prey were absent (Fig 1). The growth rate of strain CCMP1393 cultured in continuous light after grazing activity ceased was slow but significant at 0.14 d^-1^, and comparable to its growth rate in the light with no HKB added (0.11 d^-1^). Therefore, a pure phototrophic nutrition did not allow an increase in abundance as fast as a mixotrophic nutrition, but it still provided more energy and nutrients than the minimal requirements for cell maintenance in this species. In contrast, light did not enhance the growth rate of strain BG-1 in the presence of prey (comparison of the left and middle panels of Fig. 1 in [13]), nor did it lead to a continued increase in population abundance after the depletion of HKB (right panel of Fig. 1 in [13]).

It is possible that strain CCMP1393 relied solely on bacteria for nutrition in the presence of both light and prey, and then switched rapidly to purely phototrophic nutrition when prey became unavailable. However, it is more likely that photosynthesis was active in the presence of prey. In fact, transcriptome results implied that strain CCMP1393 had higher photosynthetic activities when prey were present (discussed in details in the following section). Furthermore, the difference in the importance of photosynthesis to the two species can also be inferred from their pigment content as strain CCMP1393 had significantly more chlorophyll *a* per cell (108–443 fg algae^-1^) than strain BG-1 (2–59 fg algae^-1^; [13]). Moreover, strain CCMP1393 placed in constant darkness continued to increase in cellular chlorophyll *a* content, resembling the photoacclimation response of typical phytoplankton [31], while strain BG-1 kept in constant dark decreased in cellular chlorophyll *a* content [13].

### Enhancement of photosynthesis by phagotrophy for strain CCMP1393

Phagotrophy appeared to increase the photosynthetic capabilities of strain CCMP1393, as evidenced by the upregulation of > 50% of genes related to photosystems in the presence of HKB (Fig 3; right 4 columns of Table 2). Genes for antenna proteins were generally upregulated in the presence of HKB (Table 2), and HKB also promoted chlorophyll production as implied by the higher expression of genes for GluTR and ChlG in treatments with HKB (Fig 4). Indeed, cellular chlorophyll *a* content was significantly higher in cultures supplied with HKB than those not receiving HKB under the same light condition (Fig 2). Conversely, strain BG-1 and other *Ochromonas* or *Poterioochromonas* species reduced cellular chlorophyll *a* content in the presence of prey [13,32,33]. Half of the genes for antenna proteins were upregulated in Light+depleted HKB compared to Light+HKB for strain BG-1, with the remaining not differentially expressed between the 2 treatments (Fig. 2 in [13]). Therefore, in contrast to strain CCMP1393, strain BG-1 appeared to enhance its light harvesting and photosynthetic capabilities only after the depletion of prey.

It is possible that bacteria provided specific material(s) that supplemented photosynthesis in strain CCMP1393. A previous study on a marine *Ochromonas* sp. that is also obligately phototrophic suggested that iron acquisition was one of the reasons for bacterial ingestion [11]. That study showed algae fed with iron-rich bacteria achieved comparable growth rates to those growing at high concentrations of dissolved iron. Iron acquisition through bacterial ingestion has also been suggested for the prymnesiophyte *Prymnesium parvum* since the alga downregulated its expression of iron uptake genes in the presence of bacteria [34]. Iron plays an important role in phytoplankton metabolism as it is a component of many proteins involved in photosynthetic and respiratory electron transport, chlorophyll synthesis, and other metabolic reactions [35]. Ferroportins are involved in the export of iron from the cell [36], and the upregulation of the gene coding for it in the presence of HKB (i.e. upregulated in Light+HKB vs. Light+depleted HKB or Light without HKB) for strain CCMP1393 implied the alga was taking up excess iron from HKB that had to be released. Conversely, the sole gene for iron uptake in strain CCMP1393 (a gene for low-iron inducible periplasmic protein FEA1) was upregulated in the Light+depleted HKB treatment when compared to Light+HKB. This finding is consistent with the speculation that the alga was in an iron-deficient state when it was not feeding on bacteria [37].

Phagotrophy may also aid in the photosynthesis of strain CCMP1393 by providing a surplus of CO_2_ for increased carbon fixation efficiency. Rubisco is an important enzyme for the fixation of CO_2_, but O_2_ is a competitive inhibitor of CO_2_ for the enzyme. Photorespiration of O_2_ instead of photosynthesis of CO_2_ can occur at low concentrations of CO_2_ [38]. We speculate that strain CCMP1393 may generate CO_2_ via the breakdown of bacterial carbon, which would result in an increase in intracellular CO_2_ that helps drive the reaction towards photosynthesis instead of photorespiration. Maranger *et al*. [11] made similar suggestions as they estimated 65% of bacterial carbon was converted to CO_2_ by the obligately phototrophic *Ochromonas* strain NEPCC no. 457 grazing on ^14^C labelled bacteria. In our study, the majority of genes exclusively related to the Calvin cycles were upregulated in the presence of HKB (right 4 columns of Table 2), especially in the Light+HKB treatment compared to Light without HKB, where 5 of 6 Rubisco genes were upregulated. A strong reliance of photosynthesis (including the light-dependent reaction) could explain the inability of strain CCMP1393 to increase in population abundance in the dark despite continued grazing on bacterial prey.

The carbon concentrating mechanism (CCM) of chrysophytes has not been well studied, and current results indicate freshwater species have no CCM and rely on diffusive CO_2_ entry [39–41]. Such a mechanism of acquiring inorganic carbon may be sufficient for freshwater species living in streams or lakes where the CO_2_ concentrations are typically higher than seawater [42], but may be insufficient for the marine strain CCMP1393 with high photosynthetic activities. Our transcriptome data indicated that the alga had genes coding for proteins related to CCMs, including carbonic anhydrase (for a biophysical CCM) and PEPC/PEPCK etc. (for a biochemical C4 photosynthesis CCM). The presence of these enzymes alone, however, does not support the occurrence of CCMs as they have other functions in the cell. Therefore, it is unclear whether strain CCMP1393 has any CCM, and bacterial ingestion can potentially be part of a CCM for this species that has yet to be characterized.

### Comparison of carbon and nitrogen metabolic pathways between *Ochromonas* strain CCMP1393 and strain BG-1

Strain BG-1 incorporated substantial bacterial carbon via glycolysis and the TCA cycle [13]. The gene expression of strain BG-1 revealed higher glycolytic and TCA cycle activities in the Light+HKB treatment compared to Light+depleted HKB (Fig. 4 in [13]). Genes related to the conversion of succinyl-CoA to oxaloacetate in the TCA cycle, as well as the gene for PEPCK, were upregulated in the presence of HKB for strain BG-1, suggesting that carbon skeletons from the breakdown of bacterial amino acids were introduced into the TCA cycle and subsequently converted to phosphoenolpyruvate [13]. Results from a nanoSIMS study on the contribution of bacterial carbon to the carbon content of strain BG-1 confirmed that 89–99% of cellular carbon in the alga was obtained from prey [43].

On the other hand, it is unclear how, or to what degree, strain CCMP1393 may have incorporated organic carbon from bacterial prey. There was no evidence of bacterial carbon incorporation by strain CCMP1393 through glycolysis, as unidirectional genes for the utilization of glucose (e.g. glucokinase (GCK) and phosphofructokinase (PFK)) were not differentially expressed between treatments with or without HKB (Fig 5). In addition, there was no upregulation of genes related to the conversion of succinyl-CoA to oxaloacetate in the presence of HKB, and PEPCK was downregulated in treatments with HKB. Hence, strain CCMP1393 did not take up bacterial carbon through processes that occurred in strain BG-1. Genes related to PPP, an alternative pathway to glycolysis, were also upregulated in the presence of HKB for strain BG-1 but not for strain CCMP1393 (Fig 5). Instead, strain CCMP1393 upregulated genes exclusively related to the Calvin cycle in the presence of HKB (Table 2). The overall gene expression of strain CCMP1393 thus suggested that the alga relied on photosynthesis and carbon fixation in the Calvin cycle even in the presence of prey, and may not have incorporated much organic carbon directly from bacteria. Although, as previously discussed, bacterial carbon may have been respired to CO_2_ and used to fuel photosynthesis.

The expression of AMT genes was upregulated in the presence of HKB for strain CCMP1393 (Fig 6), but downregulated in similar conditions for strain BG-1 (Fig. 5 in [13]). AMTs can either import or export NH_4_^+^ across the cell membrane [44], but AMT genes upregulated in the Light+depleted HKB treatment for strain BG-1 were likely coding for AMTs that imported NH_4_^+^ into the alga as this species took up NH_4_^+^ in the absence of bacterial prey [7,43]. Unfortunately, the similarity in the AMT protein sequences between strain CCMP1393 and strain BG-1 was low (< 50% similarity), so it is unclear whether strain CCMP1393 imported or exported NH_4_^+^ when grazing on bacteria. Large variations in the sequence of AMT between the two algae may imply differences in the biochemical properties, localization, and regulation of the transporters [45].

### Ecological implications of different nutritional strategies

Environmental factors most likely played an important role in the evolution of the nutritional strategies of these 2 *Ochromonas* species. Strain CCMP1393 was isolated from surface waters of the Gulf Stream (20–25 m), an environment where bacterial abundance is generally low (~ 5x10^5^ ml^-1^; [46]). This is in agreement with the relatively low bacterial grazing rates observed for this alga (< 10 HKB alga^-1^ h^-1^) and its ability to grow, albeit at a slower rate, in the absence of bacterial prey (i.e. Light without HKB treatment; Fig 1). One might expect such an oligotrophic environment [47] to select for a mixotrophic strategy that relies equally on both phagotrophy and phototrophy, perhaps even synergy between the two nutritional modes in which bacterial grazing enhances photosynthesis. In contrast, strain BG-1 was isolated from an eutrophic freshwater pond in the Malaysia Botanical Garden [7]. Ample prey may have reduced the need for light and photosynthesis for this freshwater species. Hence, the photosynthetic capabilities of this strain only allow for prolonged survival but not an increase in population abundance when prey occur at low abundance [13].

Differences in their nutritional strategies result in different functional roles between *Ochromonas* spp. strain CCMP1393 and strain BG-1. Strain CCMP1393 is a relatively inefficient grazer on bacteria (≤ 10 HKB alga^-1^ h^-1^) compared to strain BG-1 (~ 40 HKB alga^-1^ h^-1^). Instead, transcriptomic evidence suggests high photosynthetic activities even in the presence of prey for the marine species, and thus a potential net primary producer role in the community. On the other hand, strain BG-1 contributes little to primary production. It releases excess nutrients from its prey when grazing on bacteria, but may become a competitor for nutrients when it is not feeding on prey.

Our study demonstrated how resource availability and the difference in the nutritional strategies and physiologies of two mixotrophic congeners affect their metabolism. As more attention is placed on studying and modelling mixotrophy, it is important to recognize the disparity among mixotrophic strategies, and how environmental factors can affect the functional roles of mixotrophs in contrasting ways, even for congeneric species.

## Supporting information

S1 Table The final concentration of components in the modified K media. (DOCX)

S2 Table The abbreviations used in this study and the full names of enzymes involved in major carbon metabolic pathways, major nitrogen metabolic pathways, and tetrapyrrole synthesis. (DOCX)
