# Supplementary material for: A tale of two mixotrophic chrysophytes: Insights into the metabolisms of two Ochromonas species (Chrysophyceae) through a comparison of gene expression
Source: PLoS One. 2018 Feb 13;13(2):e0192439. doi: 10.1371/journal.pone.0192439 (PMC5811012; doi:10.1371/journal.pone.0192439)
Supplement: S1 Table — (DOCX) [file pone.0192439.s001.docx]

**Table S1. The final concentration of components in the modified K media.**

| **Component** | **Final concentration in media (μM)** |
| --- | --- |
| Tris-base (pH 7.2) | 1000 |
| NaH_2_PO_4_• H_2_O | 36.2 |
| NH_4_Cl | 50 |
| NaNO_3_ | 882 |
| Na_2_ β-glycerophosphate | 10 |
| Na_2_EDTA•2H_2_O | 12 |
| FeCl_3_•6H_2_O | 12 |
| MnCl_2_•4H_2_O | 0.9 |
| ZnSO_4_•7H_2_O | 0.08 |
| CoCl_2_•6H_2_O | 0.05 |
| CuSO_4_•5H_2_O | 0.01 |
| Na_2_MoO_4_•2H_2_O | 0.08 |
| H_2_SeO_3_ | 0.01 |
| NiSO_4_•6H_2_O | 0.01 |
| Na_3_VO_4_ | 0.01 |
| K_2_CrO_4_ | 0.01 |
| Thiamine | 0.3 |
| Biotin | 0.002 |
| Cyanocobalamin | 0.0004 |
| Soil extract | 1 ml/L |
