# Supplementary material for: A tale of two mixotrophic chrysophytes: Insights into the metabolisms of two Ochromonas species (Chrysophyceae) through a comparison of gene expression
Source: PLoS One. 2018 Feb 13;13(2):e0192439. doi: 10.1371/journal.pone.0192439 (PMC5811012; doi:10.1371/journal.pone.0192439)
Supplement: S2 Table — (DOCX) [file pone.0192439.s002.docx]

**Table S2. The abbreviations used in this study and the full names of enzymes involved in major carbon metabolic pathways, major nitrogen metabolic pathways, and tetrapyrrole synthesis.**

| **Pathway** | **Abbreviation** | **Full name** |
| --- | --- | --- |
| Tetrapyrrole synthesis | ChlG | Chlorophyll synthase |
|  | FeCH | Ferrochelatase |
|  | FDBR | Ferredoxin-dependent bilin reductase |
|  | GluRS | Glutamyl-tRNA synthetase |
|  | GluTR | Glutamyl-tRNA reductase |
|  | HO | Heme oxygenase |
|  | MgCH | Magnesium chelatase |
| Major carbon metabolic pathways | ALD | Fructose-bisphosphate aldolase |
|  | CS | Citrate synthase |
|  | FBP | Fructose-1,6-bisphosphatase |
|  | GCK | Glucokinase |
|  | OGDC | Oxoglutarate dehydrogenase complex |
|  | PDC | Pyruvate dehydrogenase complex |
|  | PEPC | Phosphoenolpyruvate carboxylase |
|  | PEPCK | Phosphoenolpyruvate carboxykinase |
|  | PFK | Phosphofructokinase |
|  | PGI | Phosphoglucose isomerase |
|  | PK | Pyruvate kinase |
|  | PPDK | Pyruvate, phosphate dikinase |
| Major nitrogen metabolic pathways | AMT | Ammonium transporter |
|  | ARG | Arginase |
|  | ASL | Arginosuccinate lysase |
|  | ASS | Arginosuccinate synthase |
|  | GLDH | Glutamate dehydrogenase |
|  | GOGAT | Glutamine oxoglutarate aminotransferase |
|  | GS | Glutamine synthetase |
|  | OTC | Ornithine transcarbamylase |
|  | unCPS | Urea ammonium carbamoyl phosphate synthase |
|  | URE | Urease |
